# Highly Efficient and Reusable Denitrogenation Adsorbent Obtained by the Fluorination of PMA-MIL-101

**DOI:** 10.1021/acsomega.3c04670

**Published:** 2023-08-19

**Authors:** Zhe Zhao, Qing-He Yang, Hui-Feng Li, Meng-Ya Zong, Dan-Hong Wang

**Affiliations:** † TKL of Metal and Molecule Based Material Chemistry, School of Materials Science and Engineering, Nankai University, Tianjin 300350, China; ‡ Sinopec Research Institute of Petroleum Processing Co. Ltd., 18, Xueyuan Road, Haidian District, Beijing 100083, China

## Abstract

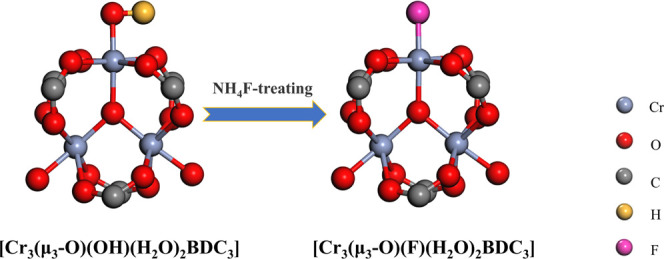

A simple but efficient strategy to improve the ability of adsorptive denitrogenation (ADN) of MIL-101(M101) was studied by the in situ encapsulation of phosphomolybdic acid (PMA) and the subsequent purification of the as-synthesized product by the NH_4_F solution. After the NH_4_F treatment, the vast majority of PMA was removed, loss of organic ligand (BDC) was observed, and the fluorination of the hydroxyl group in the M101 structure occurred. The ADN activities of the Cr-MOF matrix composites before and after fluorination were studied in detail. The rest of PMA interacts strongly with M101 and assists the ADN activity. Coordination unsaturated metal sites (CUS) in M101 are formed after fluorination and also contribute to ADN activity. Further, fluoride anions replace most of the hydroxide groups in M101, which can promote the ADN of quinoline (QUI) and indole (IND) through an acid–base interaction and N-atom coordination with the CUS in M101. P-M101-F 5% exhibits the highest adsorptive capacity and excellent regeneration ability. Special emphasis in this work is placed on structure modulation (including PMA doping, CUS creation, and fluorination) of M101 for enhancing ADN activity, which provides a useful scaffold for future research in the rational design of MOF-based ADN catalysts.

## 1 Introduction

Studies have found that about one-fifth of deaths are caused by air pollution from fossil fuels.^1^ The main harmful compounds in fossil energy are sulfur-containing compounds and nitrogen-containing compounds.^2−5^ The nitrogen compounds in fuel have a serious inhibitory effect on the consequent hydrodesulfurization process.^6,7^ Thus, the industry needs an efficient, energy-saving, and environmentally friendly denitrogenation technology. Compared with the industrialized hydrodenitrogenation process with large hydrogen consumption and high cost, adsorptive denitrogenation (ADN) not only shows a high efficiency of denitrogenation but also solves the problems of large equipment investment and high operating cost.^8^ Recently, more and more attention has been paid to this research field.^9−12^

As a new type of porous functional material, metal–organic frameworks (MOFs) are constructed by metal ions or metal cluster units and organic ligands.^13,14^ MOFs have the characteristics of large surface area, porosity,^15^ and high crystallinity.^16,17^ There are regular and dense adsorption active sites in the pores of MOFs. Therefore, MOFs are widely used to adsorb nitrogen compounds in fuel. Jhung et al.^18−20^ showed that M101 functionalized both on metal and ligand can effectively adsorb quinoline (QUI) and indole (IND). M101 is a promising MOF adsorbent in the research area of ADN. Design and development for more efficient functionalized M101 are meaningful for ADN processes.

Fluorine has been extensively studied because of its high electronegativity and hydrophobicity.^21−23^ However, fluorine interacting with M101 still has some functions that are not yet clear. During the hydrothermal reaction, fluorine seems to provide a strong interaction with chromium octahedral motif and effectively promote the formation of M101.^24^ In the MOF research, labile organic ligands linked with metal sites can be eliminated to generate coordination unsaturated metal sites (CUS),^25^ which also present high catalytic activity due to strong interaction with organic reactants through an acid–base interaction or π-complexation.^26^

**Scheme 1:**
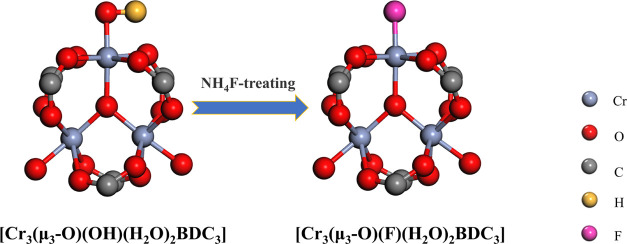
Strategy for Synthesizing M101-F from M101 by Anion Postreplacement

In this study, encapsulation of phosphomolybdic acid (PMA) into M101 cavities first results in the loss of organic ligands. Then, purification of the as-synthesized product by NH_4_F can further create ligand defects, which resulted in the production of CUS. A large amount of PMA was eliminated and the free uncoordinated terephthalate acid in the M101 pores was eluted by NH_4_F simultaneously^16^ to obtain higher surface area and pore volume. Fluoride anions can also be exchanged into M101 structural units to form F–Cr bonds with the formula Cr_3_(μ_3_-O)(F/OH)(H_2_O)_2_BDC_3_ (M101) (Scheme 1). In this work, the effects of PMA, ligands defect, and fluoride (F–Cr) on denitrogenation were deeply investigated. Theoretical calculations are also used to study the adsorption energy between the nitrogen compounds and the adsorbent. Fluorinated M101 might be a potential adsorbent for the purification of nitrogen compounds in the fuel.

## 2 Experimental Section

### 2.1 Preparation of Adsorbents

#### 2.1.1 Synthesis of M101, M101-F

The synthesis method of M101 has been slightly modified from the previously reported procedure^27,28^ and was synthesized by conventional electric heating. The specific synthesis method is shown in the Supporting Information.

#### 2.1.2 Syntheses of P-M101 *x*% and P-M101-F *x*%

Except that PMA was added to the synthetic solution, P-M101 *x*% has been synthesized by the same method as M101. The adsorbent was acquired by adding different PMA contents. The samples were defined as P-M101 *x*% (*x* = 2, 5, 10, 20 indicating the content of adding PMA). For example, P-M101 2% means that the content of adding PMA is 2% of the total mass content of Cr(NO)_3_·9H_2_O + BDC. After obtaining P-M101 *x*%, 0.5 g of P-M101 *x*% was dispersed in 75 mL of deionized water with 30 mmol NH_4_F and stirred at 60 °C for 10 h. After cooling, traces of NH_4_F are removed from the precipitate by washing three times with hot water (60 °C). Finally, the solid was dried in a vacuum at 150 °C for 12 h to acquire P-M101-F *x*%.

### 2.2 Characterization

The powder X-ray diffraction (PXRD) patterns were recorded in the range 3–50° at a scanning speed of 10 deg/min and a step size of 0.02 deg with a Rigaku Miniflex 600 diffractometer with Cu Kα radiation (λ = 0.154178 nm). The Fourier transform infrared (FT-IR) spectrum was recorded on 273k Bruker tensor 37. Energy-dispersive X-ray spectroscopy (EDS) elements mapping images were observed under a JEM-2800 microscope. X-ray photoelectron spectroscopy (XPS) results were obtained from Thermo Scientific K-α. The ^1^H solid-state NMR experiments were performed on a 400mhz Bruker Avance III HB spectrometer using a Bruker 4 mm ^1^H/^31^P–^15^N CPMAS probe. Thermo Fisher iCAP PRO(OES) adopted a thermal science spectrum blue ICP-OES spectrometer. The thermal stability of the adsorbent was analyzed by thermogravimetric analysis (TGA). The Brunauer–Emmett–Teller (BET) results showed nitrogen adsorption–desorption isotherms, N_2_ was used as an adsorbate at 77.4K, and the degassing condition was 150 °C for 12 h.

Innovatively, potentiometric acid–base titration has been used to seek the amount of μ3-OH and Cr–OH. Potentiometric acid–base titration was performed with a ZDJ-4B potential titrator. Before sample preparation, the sample was dried at 150 °C in a vacuum for 12 h and then ground into finer powder. Then, 25 mg of the sample was weighed and added into 50 mL of 0.01m NaNO_3_ solution for equilibrium for 12 h. Then, the pH of the solution was adjusted to 3.00 using 0.1 M HCl. During the titration process, the pH of the prepared solution was titrated to 10.5–11.0 with a 0.05 M NaOH solution at a titration rate of 0.020 mL/min. In order to show the equivalent point better, the titration curve is the first derivative and the corresponding equivalent points will appear on the first derivative curve.

### 2.3 Adsorptive Denitrogenation Test

First, a model oil with 500 ppm of quinoline (QUI) and 500 ppm of indole (IND) was prepared by dissolving QUI and IND in *n*-octane. Then, 10 g of model oil and 20 mg of adsorbent were added to a round-bottom flask. The mixture was stirred continuously at room temperature for 1 h. After the reaction was completed, the mixture was centrifuged and the supernatant was used for adsorption nitrogen removal tests.

The nitrogen content in the experiment was measured by a high-performance liquid chromatography (HPLC) Agilent 1200 series chromatographic column analyzer. A C-18 chromatographic column with a length of 250 mm, a diameter of 4.6 mm, and a particle size of 5 μm was used. The test conditions were as follows: the initial mobile phase was 40% deionized water and 60% methanol and the flow rate was 0.6 mL/min.

### 2.4 Calculation Detail

The exact role of fluorine in the synthesis and application of MIL-101 has not been clarified. To explore the influence of fluoride anions on the ADN performance of adsorbent, the M101 model was established, and the adsorption energies of QUI and IND with M101 and M101-F cluster forming the Cr–F bond from the original Cr–OH bond were calculated theoretically (Scheme 1). The rationality of calculating the adsorption energy of similar metal cluster structures has been reported in the relevant literature.^29^ The Vienna ab initio package (VASP) has been employed to perform all of the density functional theory (DFT) calculations within the generalized gradient approximation (GGA) using the Perdew–Burke–Ernzerhof (PBE) formulation. The adsorption energy (*E*_ads_) of adsorbate A was defined as eq 1
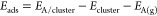
1where *E*_A/cluster_, *E*_cluster_, and *E*_A(g)_ are the energy of adsorbate A adsorbed on the cluster, the energy of clean cluster, and the energy of isolated A molecule in a cubic periodic box with a side length of 20 Å and a 1 × 1 × 1 Monkhorst–Pack *k*-point grid for Brillouin zone sampling, respectively.

## 3 Results and Discussion

### 3.1 Characterization of Adsorbents

**Figure 1:**
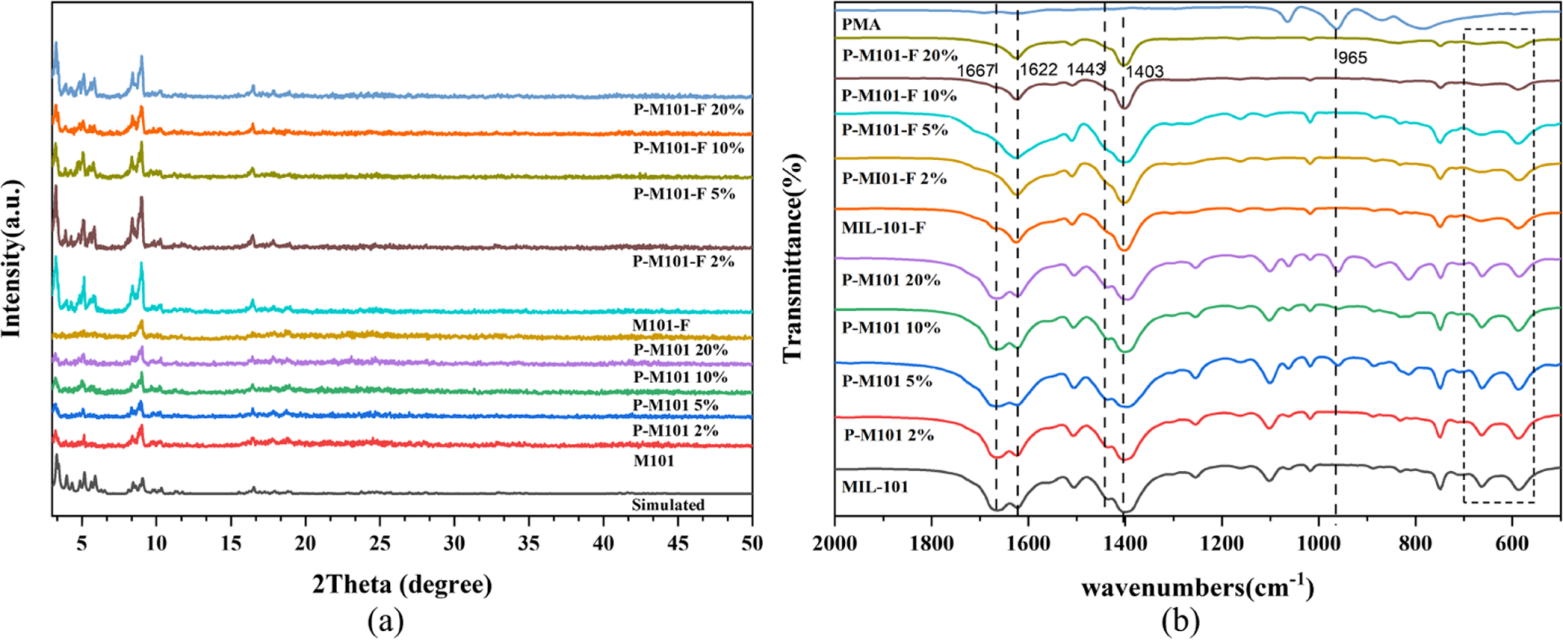
(a) XRD pattern of the samples. (b) FT-IR spectrum of the samples.

Figure 1a shows the XRD patterns of M101, M101-F, P-M101 *x*%, and P-M101-F *x*%. It can be seen that the peak intensity and peak position of the prepared samples are consistent with those of simulated MIL-101, which proves that all samples were successfully synthesized with the MIL-101 structure. No new characteristic peak appears in P-M101 *x*% composites after PMA addition, and no new characteristic peak appears in P-M101-F *x*% composites after purification by NH_4_F, which proves that PMA was highly dispersed in P-M101 *x*% and P-M101-F *x*%. After further soaking in NH_4_F, the diffraction peak intensity of P-M101-F *x*% composites increased, indicating that the crystallinity of P-M101-F *x*% became good and the grain size increased. At the same time, the NH_4_F modification will not change the M101 structure of the composites. By assuming 100% crystallinity for the simulated pattern, taking this as a reference, the crystallinity of M101-F and all P-M101-F *x*% composites obtained in this study is similar, and the crystallinity range is 40–47%. Among them, the crystallinity of M101-F and P-M101-F 5% is 44.31 and 44.32%, respectively, which proves that the crystallinity of the prepared samples is good. This result was obtained according to X’pert software outputs.

FT-IR test was applied to clarify the role of NH_4_F modification on the M101-F and P-M101-F *x*% composites. As shown in Figure 1b, the FT-IR spectrum of pure PMA has characteristic peaks at 1064, 961, 867, and 782 cm^–1^. which are assigned to the stretching vibrations of P–O_a_, Mo = O_d_ (terminal oxygen), Mo–O_b_–Mo, and Mo–O_c_–Mo (bridging oxygen), respectively.^30^ The FT-IR spectra of P-M101 *x*% show the characteristic peak at 961 cm^–1^ corresponding to Mo = O_d_ of PMA, and the intensity of this peak increases with the PMA content. It is worth noting that after NH_4_F modification, the characteristic peak of PMA in P-M101-F *x*% weakened or even disappeared, which may indicate that PMA was eluted by NH_4_F soaking to some extent. The amounts of Mo loading for P-M101-F *x*% composites are shown in Table S1, and the Mo content in P-M101-F *x*% composites is 0.233–2.053 wt %, which is much less than the initial amount used in the synthesis. At the same time, NH_4_F soaking eliminated unreacted terephthalic acid (BDC). In fact, NH_4_F and BDC are easily reacted to obtain ammonium terephthalate, which is easily soluble in water and thus can be removed.^24^ From Figure 1b, we can find that the peak intensities of free carboxylic acids at the positions of 1666 cm^–1^ (stretching vibration of C=O in the carboxyl group –COOH) and 1444 cm^–1^ (deformation vibration of –OH in –COOH) disappear after NH_4_F soaking, indicating that the uncoordinated carboxylic acids in M101/P-M101 pores have been washed away by NH_4_F. The two strong bands of the samples at 1620 and 1404 cm^–1^ can be attributed to typical νas (–COO^−)^ and νs (–COO^–^) of carboxylate, respectively. The positions of 662 and 584 cm^–1^ belong to the O–Cr–O absorption peaks of BDC connected with Cr.^31,32^ It is worth noting that one of the two vibrational strengths of O–Cr–O was weakened after NH_4_F modification. This result suggests that coordination around the Cr(III) centers may change.

**Figure 2:**
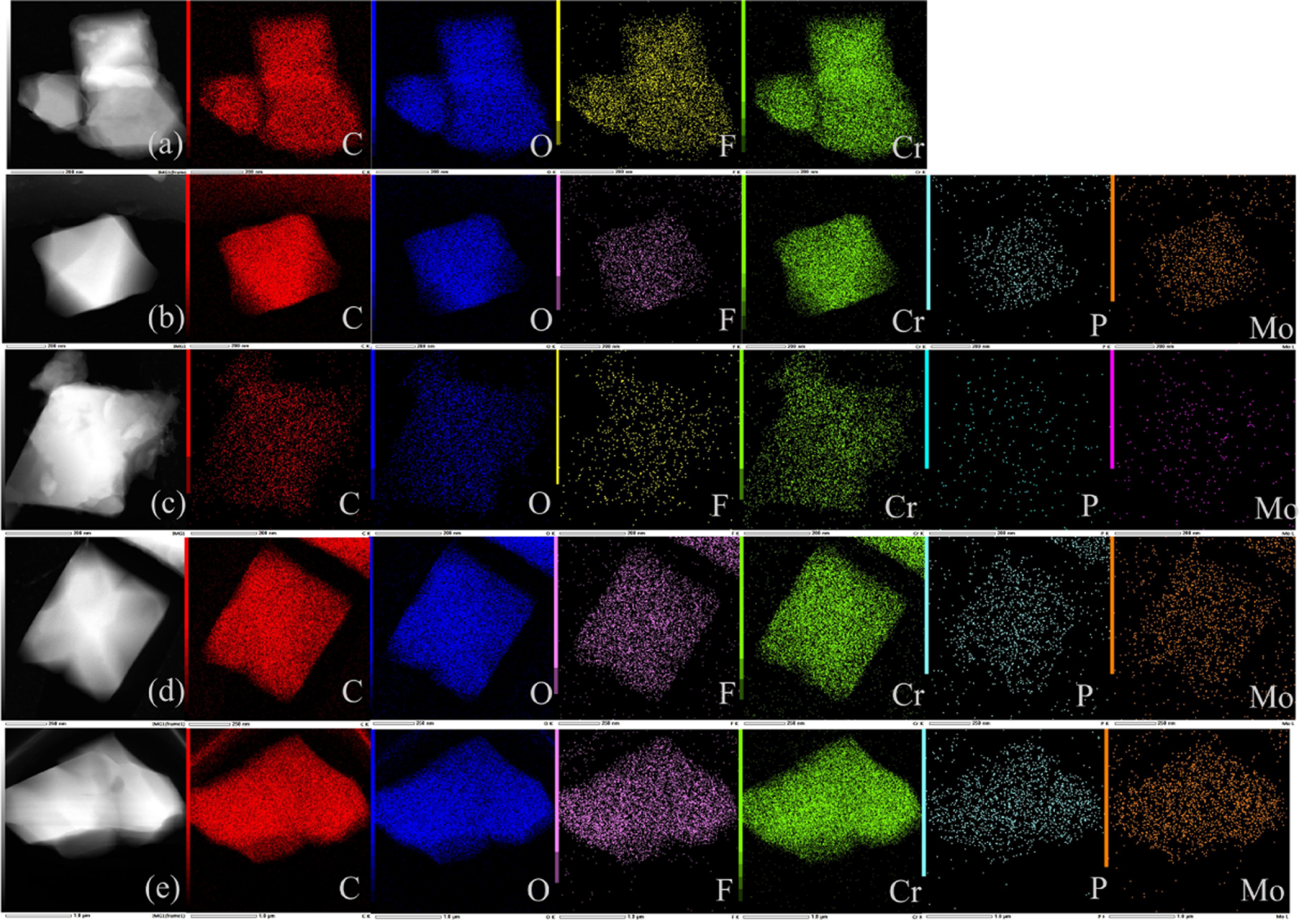
EDS element mapping images of (a) M101-F, (b) P-M101-F 2%, (c) P-M101-F 5%, (d) P-M101-F 10%, and (e) P-M101-F 20%.

**Figure 3:**
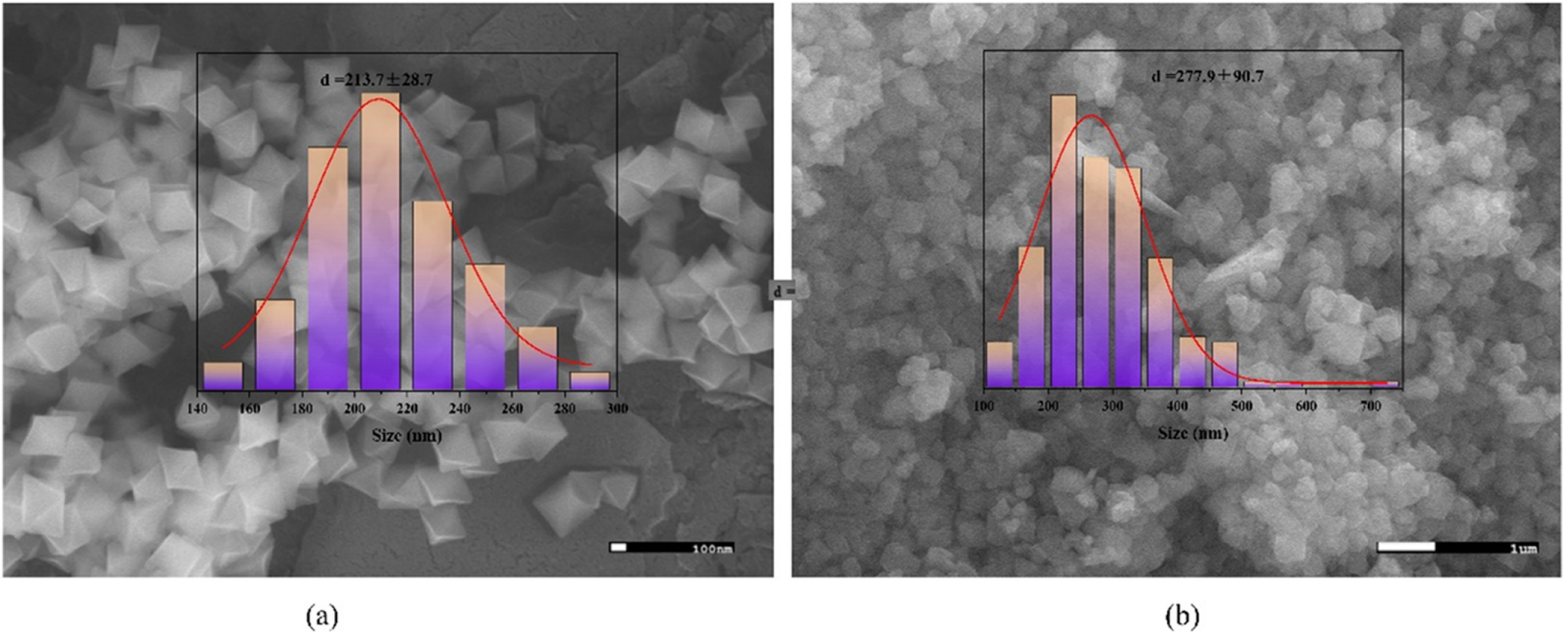
SEM images and particle size distribution of (a) M101-F and (b)P-M101-F 5%.

It can be clearly seen by EDS element mapping images that the prepared composites exhibit an octahedral nanoparticle shape similar to M101, such as in Figure 2. Through EDS mapping images of P-M101-F *x*%, it can be seen that the elements such as C, O, F, Cr, P, and Mo are all evenly distributed. All characteristics indicate that PMA has been highly dispersed in P-M101-F. As shown in Figure 3, the particle size was calculated from scanning electron microscopy (SEM) images. The particle size of about 200–300 nm was observed for the composites.

**Figure 4:**
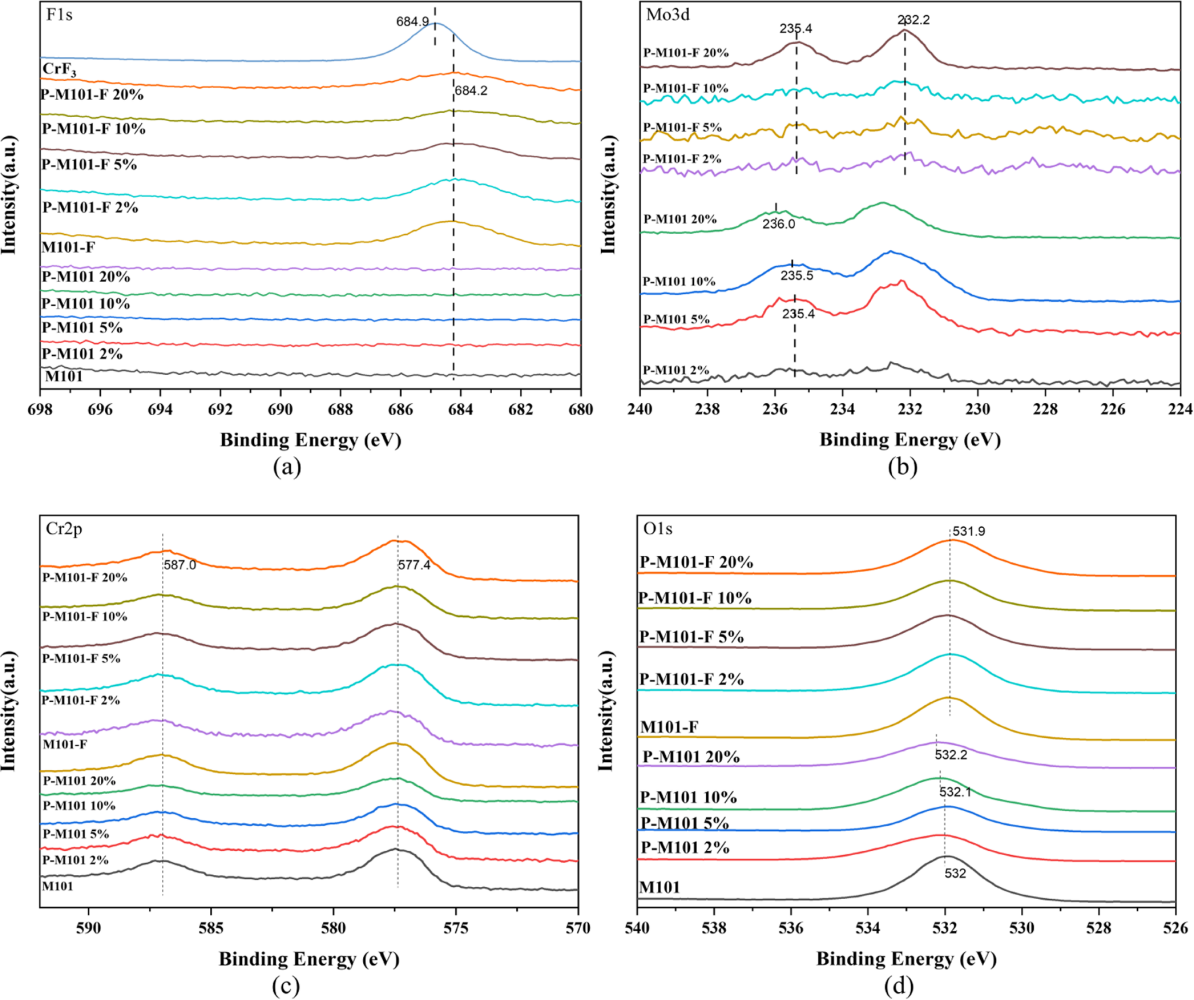
XPS spectra of the obtained adsorbents: (a) F 1s, (b) Mo 3d, (c) Cr 2p, and (d) O 1s sites.

The XPS results were collected to further analyze the chemical valence and elemental composition of the composites. As shown in Figure S1, C—C (284.8 eV) was used to correct the binding energy (BE) data. As shown in Figure 4a, compared with M101 and P-M101 *x*%, M101-F and P-M101-F *x*% show new peak at 684.3 eV, which is attributed to the F–Cr bond. This result can prove that fluoride anions replace the hydroxyl group to some extent by the anion postreplacement method^22^ with the formula of Cr_3_(μ_3_-O)(F/OH)(H_2_O)_2_BDC_3_ (M101). For Mo 3d in Figure 4b, the characteristic peaks at 232.2 and 235.4 eV in P-M101-F *x*% are related to Mo(VI) 3d_5/2_ and Mo(VI) 3d_3/2_. It is worth noting that the characteristic peaks of Mo 3d in P-M101 *x*% shift toward the direction of high BE with the increase of the PMA amount and Mo 3d BEs decreased after fluorine modification. This result verifies that PMA is loaded and interacts with M101 successfully. According to Figure 3c, the characteristic peaks at 587 and 577.4 eV remain unchanged and can be easily assigned to Cr(III) 2p_3/2_ and Cr(III) 2p_1/2_ in M101/P-M101 *x*% and M101-F/P-M101-F *x*%, respectively. The BE variation trend of the O 1s spectra is similar to that of the Mo 3d spectra, the characteristic peaks of O 1s in P-M101 *x*% shift toward the direction of high BE with the increase of the PMA amount and the decrease after fluorine modification, indicating that the introduction of fluorine results in lower BEs of Mo 3d and O 1s for PMA and implies an interaction between PMA and fluoridized M101. As shown in Figure S2, the ^1^H NMR spectrum was analyzed. M101 had the –OH peak and H_2_O peak, and the hydroxyl peak attenuated in the NMR spectra of M101-F or P-M101-F *x*%. It is proven that the replacement of fluorine reduces the amount of Cr–OH in M101-F or P-M101-F *x*%.

**Figure 5:**
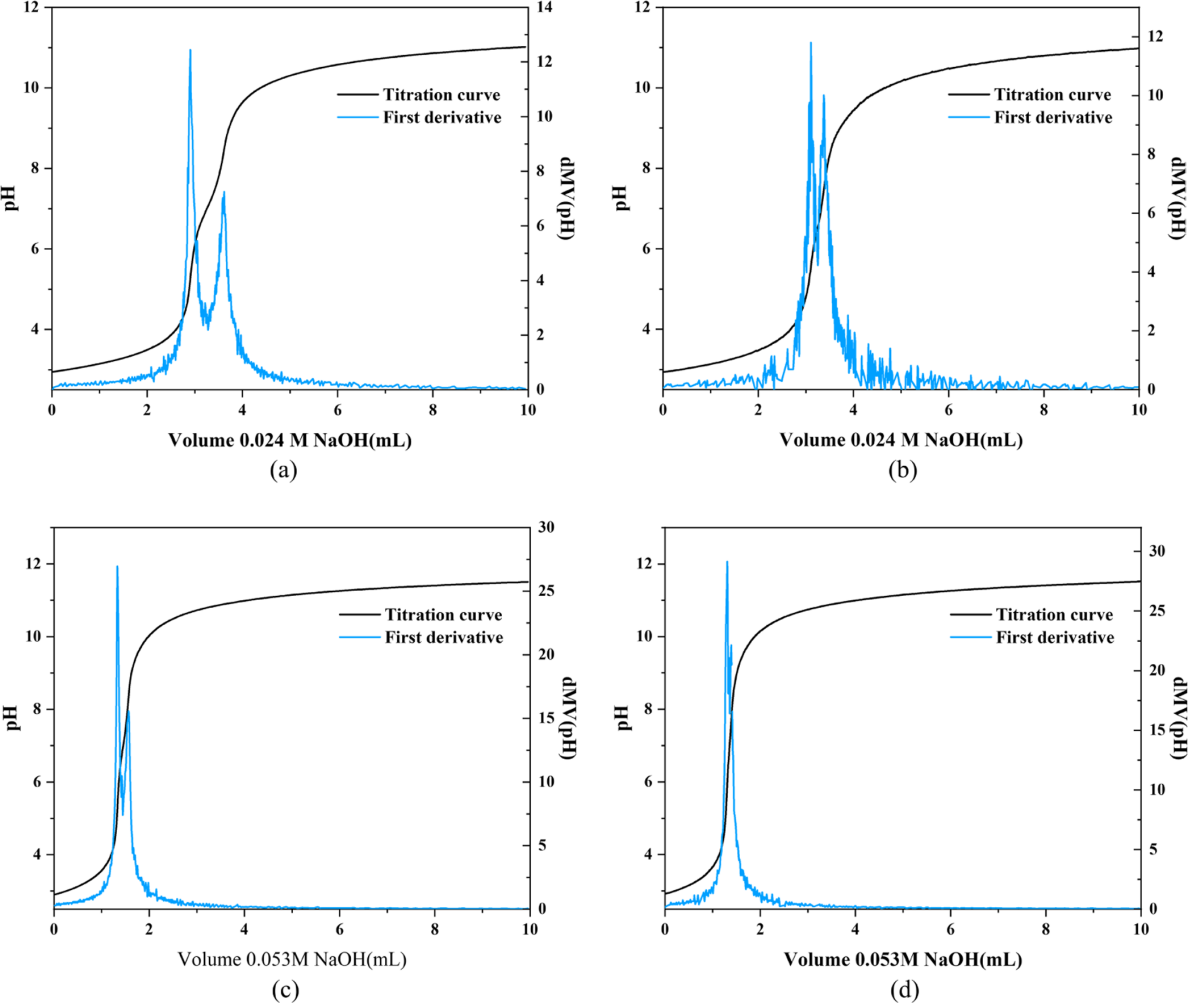
Acid–base titration curve and the corresponding first derivative curve for (a) M101, (b) M101-F, (c) P-M101 5%, and (d) P-M101-F 5%.

In order to verify the changes in Cr–OH more accurately, a potentiometric acid–base titration method was used to analyze the samples. The potentiometric acid–base titration curve and first derivative curve for the samples are shown in Figure 5. The first derivative of the titration curves for all samples shows two distinct peaks. The p*K*_a_ values for each jump point are listed in Table 1. According to the value of p*K*_a_, the first jump point (p*K*_a1_ ≈ 3.3) can be attributed to the proton of μ_3_–OH by the protonation of M101, while the second jump point (p*K*_a2_ ≈ 7) can be assigned to Cr–OH at the end of the Cr. It can be approximated that the two kinds of protons are titrated by NaOH in turn. Therefore, the amount of NaOH consumed between the two jump points (*n* = *n*_2_ – *n*_1_) is positively correlated with the moles of Cr–OH. It can be easily seen that *n* (M101-F) is that in M101. Similarly, the amount of Cr–OH in P-M101-F *x*% is less than that in P-M101 *x*%. This result, together with the NMR spectrum, strongly demonstrates the reduction of the amount of Cr–OH resulting from NH_4_F soaking with F–Cr bonding.

**Table 1:** p*K*_a_ of μ3-OH and Cr–OH in Different Samples

	p*K*_a1_	p*K*_a2_	Cr–OH *n*_2_ – *n*_1_ (mol*10^–6^)
M101	3.27	7.02	17.02
M101-F	3.30	6.50	6.41
P-M101 5%	3.22	6.63	12.58
P-M101-F 5%	3.27	6.92	4.38

The content of fluoride was tested, as shown in Table 2. Ion chromatography analysis showed the change of the fluoride content from 0.075 wt % for M101 and 0.128 wt % for P-M101 5% to 1.853 wt % for M101-F and 1.48 wt % for P-M101-F 5%, respectively. This result indicates that fluoride is introduced in M101. Combined with the existing study,^22^ XPS, ^1^H NMR, potentiometric acid–base titration, and ion chromatography results show that fluoride successfully replaces hydroxyl by anion postreplacement, which will change the chemical environment of MIL-101 Cr.

**Table 2:** Ion Chromatography Result for Different Samples

sample	F content (wt %)
M101	0.075
M101-F	1.853
P-M101 5%	0.128
P-M101-F 5%	1.482

According to nitrogen adsorption–desorption isotherms, the BET surface area and pore size distribution curves were obtained. As shown in Table 3 and Figure S3, all of the materials are assigned the I type adsorption–desorption isotherms, indicating that the materials have a microporous structure. The BET specific surface area of M101 is 2219.4 m^2^/g, which is larger than that of P-M101 *x*% and the pore volume is 1.05 cm^3^/g. With the increase of the PMA content, the specific surface area and pore volume of P-M101 *x*% gradually decreased, which also proved that PMA was successfully introduced into the initial M101 channel. However, the specific surface area and pore volume of M101-F and P-M101-F *x*% are greatly increased compared with those of the corresponding M101 and P-M101 *x*%, which proves that NH_4_F removed some PMA and the unreacted BDC in the channels, as proved by IR results (Figure 1b). Thanks to the fluorination of P-M101, the accessible channels of P-M101-F *x*% are increased,^33^ which means some structure is stretched out.

**Table 3:** BET Surface Area and Pore Volume of Samples

sample	*S*_BET_ (m^2^/g)	*V*_pore_ (cm^3^/g)
M101-F	2874.3	1.36
P-M101-F 2%	2885.2	1.26
P-M101-F 5%	2720.2	1.31
P-M101-F 10%	3095.5	1.38
P-M101-F 20%	2818.6	1.14
M101	2219.4	1.05
P-M101 2%	2155.7	1.02
P-M101 5%	1779.1	0.91
P-M101 10%	1697.6	0.73
P-M101 20%	737	0.32

**Figure 6:**
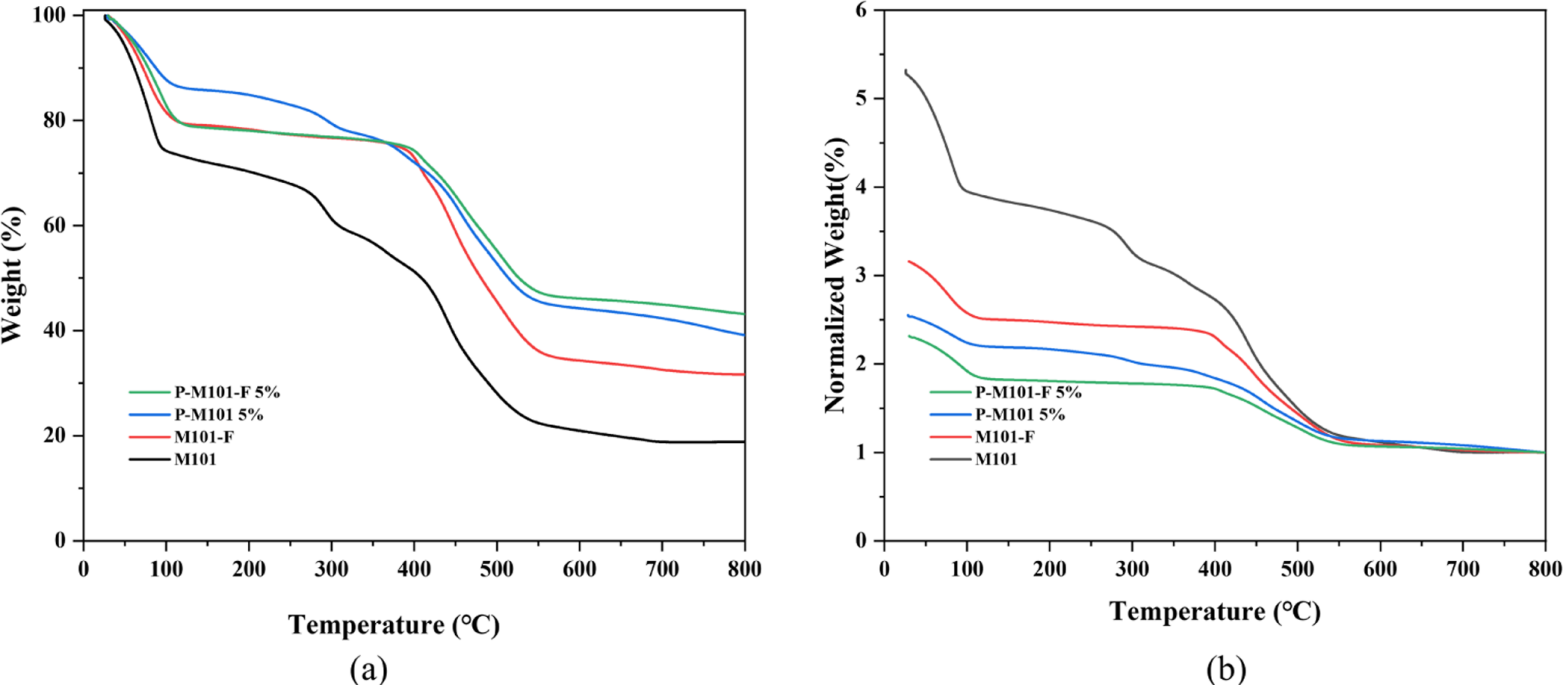
TGA curves of different samples. (a) TGA curve of the samples. (b) Thermogravimetric loss curve normalized with the minimum value.

TGA curves of the samples prove the influence of missing linkers, as shown in Figure 6a. The mass loss of M101 and P-M101 5% is mainly divided into four stages. Evaporation of volatile solvent (such as H_2_O), which is added during the hydrothermal synthesis, causes a mass loss in the 25–100 °C range. The slow mass loss in the 100–300 °C range is mainly due to the loss of DMF, which is added during the purification process. In the 300–400 °C range, the loss is due to the decomposition of disconnected terephthalic acid (unreacted BDC). The skeleton with the coordinated BDC ligands collapses in the range of 400–500 °C, resulting in a rapid mass loss. However, the mass loss of M101-F and P-M101-F 5% is mainly divided into three stages. The mass loss in the 25–100 °C range is caused by the evaporation of H_2_O. The extremely slow mass loss in the 100–400 °C range is mainly due to the evaporation of residual solvent or the disconnected BDC. The mass loss in this procedure is very low for M101-F and P-M101-F 5%, which proves that the fluorinated samples have been purified successfully. The rapid mass loss in the range of 400–500 °C is due to skeleton collapse. The framework decomposition step involves the complete combustion of the coordinated BDC linkers. The magnitude of this mass loss (when normalized as above) is inversely correlated with the defectivity of the M101 linker (CUS).^34^ TGA curves are normalized to find the proportion of BDC ligands collapsed. As shown in Figure 6b, in the range of 400–500 °C, the mass loss degree is M101 > M101-*F* > P-M101 5% > P-M101-F 5%. The result shows that the proportion of collapsible ligands is P-M101-F 5% < P-M101 5% < M101-*F* < M101. Therefore, it can be explained that NH_4_F also etched away the coordinated BDC linkers in M101. Moreover, the addition of PMA decreased the coordinated ligand of the M101 material, which resulted in more missing linker defects (CUS) after NH_4_F etching.

### 3.2 Adsorption of NCCs

**Figure 7:**
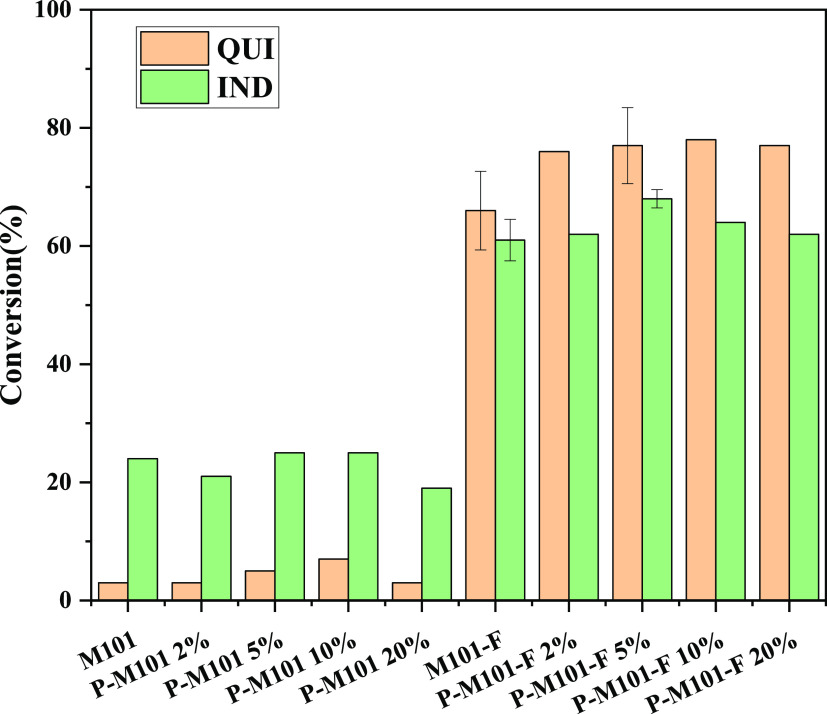
Adsorption capacity of the samples.

IND and QUI are the most typical neutral nitrides and basic nitrides in the model oil, and *n*-octane is used as the model oil. Table 4 and Figure 7 show the adsorption capacity of adsorbents for QUI and IND, and M101, P-M101, M101-F, and P-M101-F *x*% (*m*_model oil_:*m*_absorbent_ = 500:1) were chosen as adsorbents for the simultaneous adsorption of QUI and IND in model oil. Concretely, 10 g of model oil and 20 mg of adsorbent were added to a round-bottom flask. The mixture was stirred continuously at room temperature for 1 h. After the reaction, the mixture was centrifuged and filtered with a syringe filter. HPLC was used to detect the remaining nitrogen content. The adsorption performance of the adsorbent can be calculated by the formula in eq 2. Total adsorption capacity *q* = *q* (QUI) + *q* (IND).
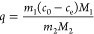
2*q* is the adsorption capacity in time 1 h (mg N/g), *C*_0_ is the initial concentration of the adsorbate (mg/g), *C_t_* is the final concentration of the adsorbate after adsorption (mg/g), *m*_1_ is the mass of the solution subjected to a single adsorption procedure (g), *m*_2_ is the mass of the adsorbent taken during a single adsorption procedure (g), *M*_1_ is molar mass of N, and *M*_2_ is the molar mass of QUI or IND.

**Table 4:** Adsorption Capacity of Samples

	adsorption	adsorption	adsorption
M101	0.72	5.93	6.65
P-M101 2%	0.76	5.24	6
P-M101 5%	1.32	6.21	7.53
P-M101 10%	1.78	6.2	7.98
P-M101 20%	0.82	4.69	5.51
M101-F	16.42	15.9	32.32
P-M101-F 2%	19.3	14.4	33.7
P-M101-F 5%	22.5	17.4	39.9
P-M101-F 10%	20.37	15.83	36.2
P-M101-F 20%	20.12	15.43	35.55

Different adsorbents have different adsorption capacities for QUI and IND, among which P-M101-F 5% has the best adsorption for QUI and IND. It can be seen from the adsorption histogram that the adsorption effect of P-M101-F *x*% on QUI increase with the increase of PMA, and the adsorption effect of IND by P-M101-F 5% is the best. It is very interesting that the adsorption activity of M101-F/P-M101-F *x*% is about 5–6 times that of M101/P-M101 *x*%, indicating that fluorination of M101 results in high adsorption capacity. The kinetics of adsorption has been was studied and is shown in Figure S4. Different from the mass of the adsorbent used previously, the adsorbent content of this adsorption reaction was changed from 20 mg to 10 mg. We can obviously see that the IND adsorption effect of P-M101-F 5% is relatively high, and the adsorption reaction reached an equilibrium in about 40 min.

### 3.3 Reusable Adsorbent

**Figure 8:**
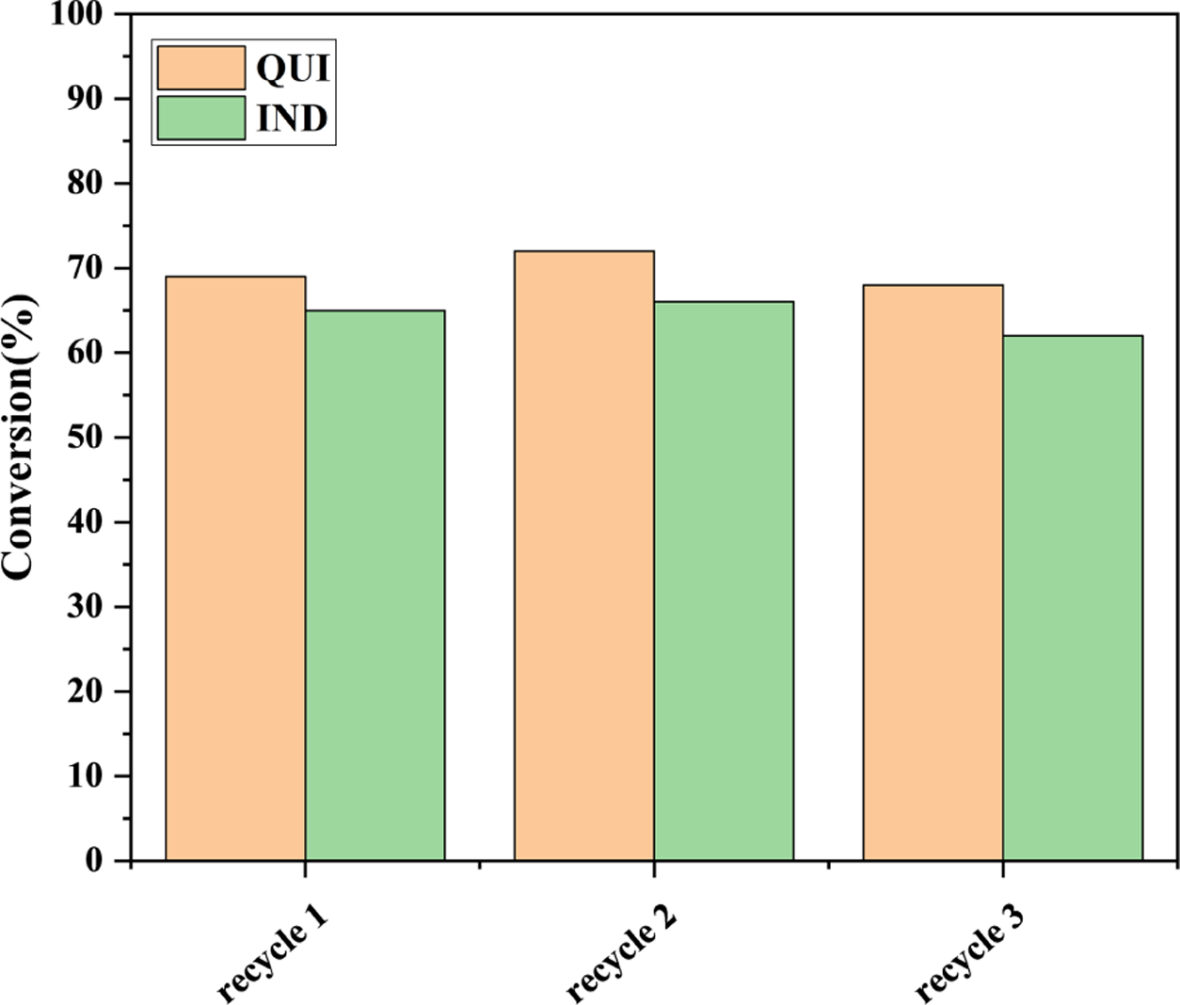
Recyclability of P-M101-F 5% for adsorption of QUI and IND from model fuel after methanol washing.

The reusability of an adsorbent is an important index to measure the industrial use value of a material. Good reusability has more potential for industrial applications with high stability. In this paper, the reusability of P-M101-F 5% was tested, and the regeneration solvent was methanol. Methanol removes NCCs from adsorbents up to 98.9% while using NCCs under ambient conditions.^35^ The adsorption capacity of QUI and IND was repeatedly tested three times. As shown in Figure 8, P-M101-F 5% has strong stability and can be regenerated by methanol. This may be due to the fact that F located at the MOFs with CUS reduces the adsorption energy of M101 with IND and QUI, which is conducive to the recycling of materials for adsorption and desorption.

### 3.4 Adsorption Mechanism

According to the above adsorption results, the adsorption capacity of M101-F and P-M101-F *x*% obtained by soaking M101 and P-M101 *x*% in NH_4_F is greatly increased. On the other hand, with similar specific surface area and pore volume, P-M101-F *x*% showed better adsorption ability for QUI and IND in model oil than M101-F *x*%, especially P-M101-F 5%. These results indicate that PMA loading, CUS, and fluorination in P-M101-F can all promote adsorption capacity. The excavated adsorption mechanism is very important for the development of adsorption technology. As we all know, the adsorption mechanism can be explained by the van der Waals force, π-complexation, acid–base interaction, coordination, and hydrogen-bond interaction.

For quinoline basic nitrides adsorbed on MOFs, the most common and important mechanisms are the acid–base interaction and coordination. Due to the existence of solitary electrons in alkaline nitrides belonging to the Lewis base, the unsaturated metal ions in MOFs (CUS) have empty d orbitals and can accept foreign electrons, which belong to Lewis acids. According to Pearson’s soft–hard acid–base theory, nitrides belonging to the hard Lewis base can interact with MOFs with CUS as a class of hard Lewis acids including Fe^3+^, Cr^3+^, and Al^3+^.

The reason for the low adsorption capacity of M101 and P-M101 *x*% adsorbents before soaking may be that the pores are occupied with residual solvent molecules and ligand residues and less exposed metal active sites (CUS). In this case, the acid–base interaction and coordination are limited. After soaking in NH_4_F, the missing BDC ligand of M101 occurred, and the encapsulation approach of P-M101 *x*% increased its amounts of CUS, which are Lewis acid sites. More Cr^3+^ is exposed, which enhances the two interactions for both acid–base interaction and coordination.

**Figure 9:**
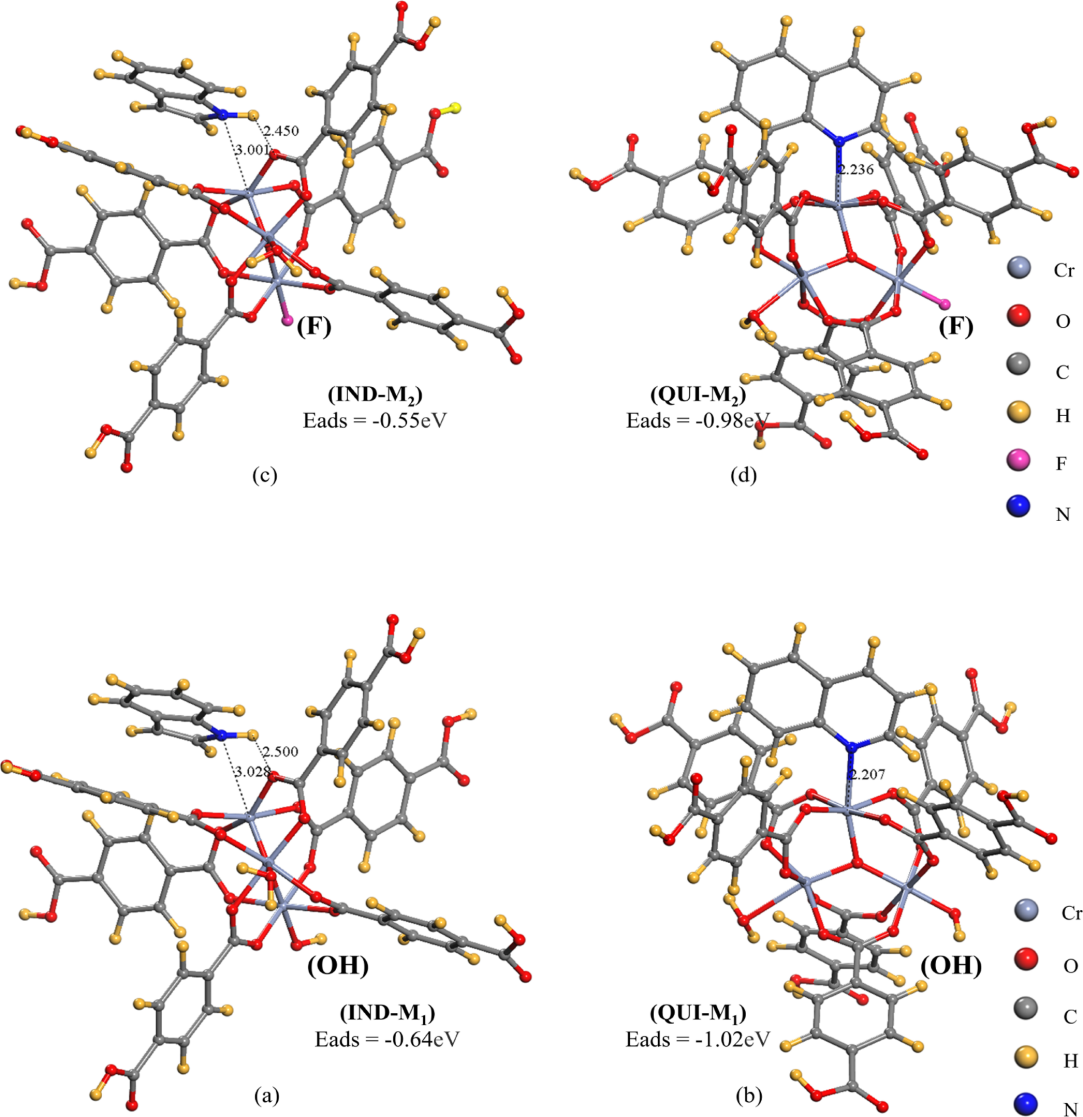
IND and QUI adsorption configurations with CUS and the corresponding *E*_ads_ of (a, b) M_1_ with [Cr_3_O (H_2_O) (C_8_O_4_H_5_)_3_OH] and (c, d) M_2_ with [Cr_3_O (H_2_O) (C_8_O_4_H_5_)_3_F].

The adsorption energies of QUI and IND at the CUS of M101 were calculated. As shown in Figure 9, the adsorption energy of QUI in M_1_ and M_2_ (−1.02, −0.98 eV) is greater than that of IND in M_1_ and M_2_ (−0.64, −0.55 eV). It may be due to the strong interaction of QUI with the unsaturated metal site Cr^3+^.^25^ In the IND-M_1_ configuration, the bond length between the IND molecule and the M site is 3.028 Å, and the H atom in IND is the closest to the O site. The N atom in IND forms a hydrogen bond through H and the O site of MOF material,^26^ indicating that the adsorption of IND at the M site can be promoted by a hydrogen bond. The N atom–metal interaction is also relatively weak due to the large angle between the IND molecule and the plane where the M site is located through a longer bond length between the N atom and the M site. The adsorption of IND is due to the combination of the hydrogen bond and the N–M interaction. When the Cr–F bond replaces the Cr–OH bond, the bond length between the N atom of IND and Cr^3+^ decreases from 3.028 to 3.001 Å, which causes IND to absorb more effectively.

## 4 Conclusions

By exploring the efficient adsorption mechanism of nitrogen compounds, subsequently more effective ADN is conducted and more environmentally friendly utilization of fuel oil is expected. In this paper, M101 was selected as the original MOF, and the method of adding moderate amounts of PMA heteropoly acids to the MOF was tried. The above experiments show that consequent purification with NH_4_F can greatly improve the performance of the adsorbents. The optimum adsorbent was P-M101-F 5%, the method of in situ synthesis by adding an appropriate amount of PMA can provide Lewis acid to MOF and avoid Mo agglomeration. Actually, by adding 10 mg of P-M101-F 5% to a 10 g solution, the adsorption capacity of QUI and IND by P-M101-F 5% reached 21.57 and 21.19 (mg N/g MOF), respectively. Combined with theoretical calculation analysis, CUS and its associated O sites were the main adsorption sites of the P-M101-F adsorbents. The selective adsorption sequence for organic nitrides is QUI > IND. The addition of PMA and soaking of NH_4_F can make M101 produce more ligand defects. The final adsorbent contains a certain amount of PMA, CUS, and fluorine, which are conducive to the adsorption of QUI and IND.

## Supporting Information Available

MOF synthesis method, different figures such as XPS spectra, ^1^H NMR spectrum, nitrogen adsorption–desorption isotherms and adsorption kinetics curve of P-M101-F 5%, different tables such as ICP-OES results for the Mo content, and comparison of the adsorption capacity of different MOFs (PDF)
