# Supplementary material for: Highly Efficient and Reusable Denitrogenation Adsorbent Obtained by the Fluorination of PMA-MIL-101
Source: ACS Omega. 2023 Aug 19;8(34):31518–28. doi: 10.1021/acsomega.3c04670 (PMC10468879; doi:10.1021/acsomega.3c04670)
Supplement: Supplementary file 1 — ao3c04670_si_001.pdf [file ao3c04670_si_001.pdf]

Supporting Information

TITLE PAGE:

Type of the Study:Article

A Highly Efficient and Reusable Denitrogenation Adsorbent Obtained by Fluorination of PMA-MIL-101

Zhe Zhao,[a] Qing-He Yang,[b]* Hui-Feng Li,[b]* Meng-Ya Zong,[a] Dan-Hong Wang[a]*

a - TKL of Metal and Molecule Based Material Chemistry, School of Materials Science and Engineering, Nankai University, Tianjin 300350, China

b - Sinopec research institute of petroleum processing co., ltd., 18, Xueyuan Road, Haidian District, Beijing 100083, China;

*Corresponding Author:

Qing-He Yang,[b]* Hui-Feng Li,[b]* Dan-Hong Wang[a]*

Phone: + 86-13821127707

E-mail: dhwang@nankai.edu.cn

A Highly Efficient and Reusable Denitrogenation Adsorbent Obtained by Fluorination of PMA-MIL-101

Zhe Zhao,[a] Qing-He Yang,[b]* Hui-Feng Li,[b]* Meng-Ya Zong,[a] Dan-Hong Wang[a]*

Experimental section

Materials: All chemical reagents used in this research were purchased and used as commercial products without further purification. N, N-dimethylformamide (DMF, 99.5%), terephthalic acid (BDC, 99%), n-octane(C_8H_{14},96%), Chromium nitrate nonahydrate ($Cr(NO)_3 \cdot 9H_2O$, 99%), QUI (C_9H_7N, 99%), IND (C_8H_7N, 99%), ethanol (C_2H_5OH, 95%), methanol (C_2H_5OH, 99.9%), ammonium fluoride(NH_4F,98%), acetonitrile(C_2H_3N,99.7%), phosphomolybdic acid hydrate(PMA).

MOFs synthesis:

M101: 4mmol $Cr(NO)_3 \cdot 9H_2O$, 4mmol p-phthalic acid (BDC), 19.2ml deionized water and 175ul HF were added to the beaker, and then ultrasonic for 10min. The mixture was transferred to a 100ml Teflon-lined autoclave and heated at the temperature of 220℃ for 8 h. The as-prepared sample was naturally cooled to room temperature. Then, the as-prepared sample was centrifuged at 10000rpm with a centrifuge and washed five times with N, N-dimethylformamide (DMF). After corresponding treatment, the green solid was collected by centrifugation and dried at a temperature of 60℃ in overnight to acquire M101.

M101-F: Furthermore, 0.5g of M101 was dispersed in 75mL of a deionized water with 30 mmol NH_4F, stirred at 60°C for 10h. After cooling, the precipitate removed traces of

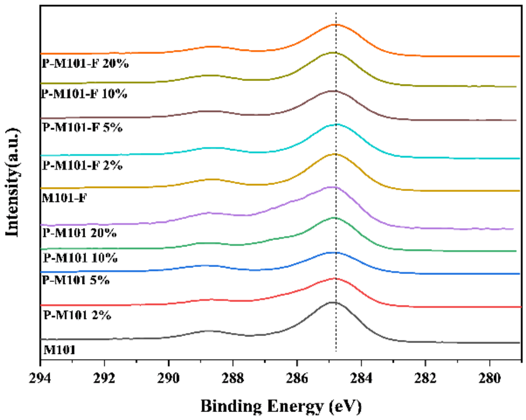

NH_4F by washing three times with hot water (60°C). Finally, the solid was dried in a vacuum at 150°C for 12h to obtain M101-F.

Figure S1. XPS spectra of C1s.

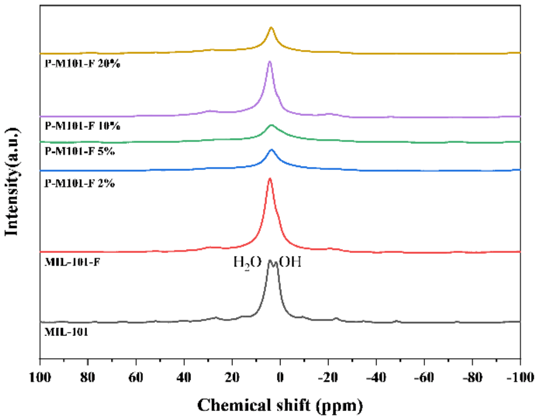

Figure S2. ^{1}H NMR spectrum of samples.

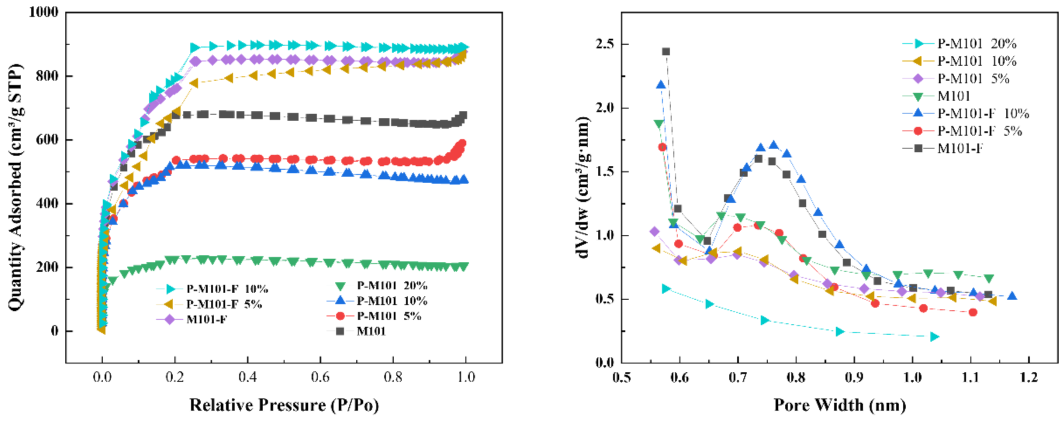

(a) (b)

Figure S3. (a) Nitrogen adsorption–desorption isotherms; (b) pore size distribution curves of the samples.

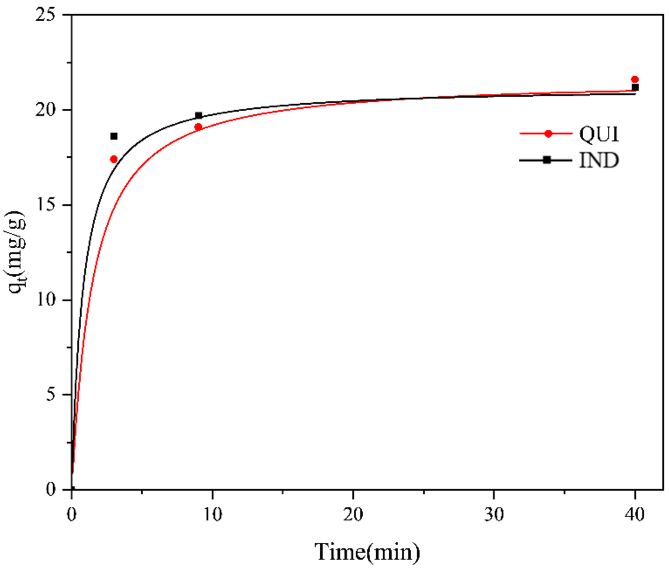

Figure S4. Adsorption capacity of P-M101-F 5%.

Table S1. ICP-OES results of the samples.

Sample	Mo content (wt.%)
P-M101-F 2%	0.233
P-M101-F 5%	0.352
P-M101-F 10%	0.580
P-M101-F 20%	2.053

Table S2. Comparison of adsorption capacity of different MOFs in 1 hour of adsorption

Conditions	Adsorbent	Adsorbate	Adsorption capacity(IND, mg/g)	Adsorption capacity(IND, mg/g)	Adsorption capacity(IND, mg/g)	Ref.
10ml n-octane+p-xylene solvent	10mg AMAS-MIL-100(Cr)	QUI+ IND+DBT	4.30	19.62	23.92	1
10ml n-octane+p-xylene solvent	10mg MIL-100(Cr)	QUI+ IND+DBT	5.02	17.89	22.91	1
10ml n-octane+p-xylene solvent	5mg 0.25% GO/ MIL101	QUI+ IND+DBT	17.57	32.52	50.09	2
10ml n-octane+p-xylene solvent	5mg $AlCl_3$(2.5%)/MIL-100(Fe)	QUI+ IND+DBT	7.65	37.72	45.37	3
20ml n-octane	5mg UiO-66-COOH	QUI+ IND	14.34	14.74	29.08	4
10ml n-octane+Isopropanol	5mg P-MIL-$125NH_2$	QUI/ IND	54.37	50.08	--	5
5ml n-octane	3mg MIL-125-VFG	QUI/ IND	47.80	41.73	--	6
20ml n-octane	5mg OC-ED-A-M101	QUI/ IND	53.78	39.29	--	7
10g n-octane	20mg P-M101-F 5%	QUI+ IND	17.40	22.50	39.90	this work

References

(1). Ahmed, I.; Hasan, Z.; Khan, N. A.; Jhung, S. H. Adsorptive Denitrogenation of Model Fuels with Porous Metal-Organic Frameworks (MOFs): Effect of Acidity and Basicity of MOFs. *Applied Catalysis B: Environmental* **2013**, *129*, 123–129.

(2). Ahmed, I.; Khan, N. A.; Jhung, S. H. Graphite Oxide/Metal–Organic Framework (MIL-101): Remarkable Performance in the Adsorptive Denitrogenation of Model Fuels. *Inorg. Chem.* **2013**, *52* (24), 14155–14161.

(3). Ahmed, I.; Jun, J. W.; Jung, B. K.; Jhung, S. H. Adsorptive Denitrogenation of Model Fossil Fuels with Lewis Acid-Loaded Metal–Organic Frameworks (MOFs). *Chemical Engineering Journal* **2014**, *255*, 623–629.

(4). Seo, P. W.; Ahmed, I.; Jhung, S. H. Adsorptive Removal of Nitrogen-Containing Compounds from a Model Fuel Using a Metal–Organic Framework Having a Free Carboxylic Acid Group. *Chemical Engineering Journal* **2016**, *299*, 236–243.

(5). Ahmed, I.; Khan, N. A.; Yoon, J. W.; Chang, J.-S.; Jhung, S. H. Protonated MIL-125-NH2: Remarkable Adsorbent for the Removal of Quinoline and Indole from Liquid Fuel. *ACS Appl. Mater. Interfaces* **2017**, *9* (24), 20938–20946.

(6). Khan, N. A.; Jhung, S. H. Phytic Acid-Encapsulated MIL-101(Cr): Remarkable Adsorbent for the Removal of Both Neutral Indole and Basic Quinoline from Model Liquid Fuel. *Chemical Engineering Journal* **2019**, *375*, 121948.

(7). Mondol, Md. M. H.; Bhadra, B. N.; Park, J. M.; Jhung, S. H. A Remarkable Adsorbent for Removal of Nitrogenous Compounds from Fuel: A Metal–Organic

Framework Functionalized Both on Metal and Ligand. *Chemical Engineering Journal* **2021**, *404*, 126491.
